# Pro-inflammatory cytokines and structural biomarkers are effective to categorize osteoarthritis phenotype and progression in Standardbred racehorses over five years of racing career

**DOI:** 10.1186/s12917-016-0873-7

**Published:** 2016-11-08

**Authors:** Andrea Bertuglia, Eleonora Pagliara, Elena Grego, Alessandro Ricci, Nika Brkljaca-Bottegaro

**Affiliations:** 1 Dipartimento di Scienze Veterinarie, Università di Torino, Largo Paolo Braccini 2, 10095 Grugliasco, Italy; 2 Clinic for surgery, orthopaedics and ophthalmology, Faculty of Veterinary medicine, University of Zagreb, Heinzelova 55, 10000 Zagreb, Croatia

**Keywords:** Post-traumatic osteoarthritis, Cross-linked C-telopeptide fragments of type II collagen, Complex oligomeric matrix protein, Interleukin-1ß, Interleukin-6, Tumour necrosis factor-α, Standardbred racehorses

## Abstract

**Background:**

Joint impact injuries initiate a progressive articular damage finally leading to post-traumatic osteoarthritis (PTOA). Racehorses represent an ideal, naturally available, animal model of the disease. Standardbred racehorses developing traumatic osteoarthritis of the fetlock joint during the first year of their career were enrolled in our study. Age-matched controls were contemporarily included. Biomarker levels of equine osteoarthritis were measured in serum and synovial fluid (SF) at baseline, and repeated yearly over the next 4 years of training (from T1 to T4). The effect of time and disease on the biomarker concentrations were analysed, and their relationship with clinical and radiographic parameters were assessed. We hypothesized that the kinetics of pro-inflammatory cytokines and structural biomarkers of joint disease would demonstrate progression of degenerative joint status during post-traumatic osteoarthritis and clarify the effect of early joint trauma.

**Results:**

The concentrations of IL1-ß, IL-6, TNF-α in the SF of PTOA group peaked at T0, decreased at T1, and then progressively increased with time, reaching levels higher than those observed at baseline starting from T3. CTXII and COMP levels were similar in PTOA and control horses at baseline, and increased in serum and synovial fluid of PTOA horses starting from T2 (serum and synovial CTXII, and serum COMP) or T3 (synovial COMP). The percentual change of TNF-α in the SF of the affected joints independently contributed to explaining the radiological changes at T3 vs T2 and T4 vs T3.

**Conclusions:**

Temporal changes of selected biomarkers in STBRs with an acute episode of traumatic fetlock OA demonstrated that long-term increased concentrations of inflammatory cytokines, type II collagen fragments and COMP, in the SF and serum, are related to PTOA. Based on the observed decrease in inflammatory merkers at T1, we hypothesize that the progression of PTOA could be effectively modulated by proper treatment strategies. Annual variations of synovial concentration of TNF-α can reliably predict radiographic progression of PTOA.

## Background

Osteoarthritis (OA) is a chronic and progressive disease that begins many years before structural changes in the whole joint are detectable. Animal models, in which hyaline articular cartilage (HAC) was experimentally impacted [1–4] or joint instability was experimentally induced [5], showed rapid progression of joint damage, at the level of the cartilage and subchondral bone [2, 6–8]. Surgical management to achieve stabilization of the knee failed to abrogate the development of post-traumatic osteoarthritis (PTOA) [9, 10], indicating that a biochemical disturbance in the HAC of the affected joint is implicated in the accelerated rate of joint degeneration. Longitudinal studies in human medicine have shown that the initial cartilage damage progresses to an established degenerative joint disease (DJD) in the hip [11] and knee joints [11, 12]. These themes of research are central to understand the optimal strategy for preventing joint degeneration [13], since both the molecular basis and the time frame of this progression are still not well-defined.

A single articular cartilage impaction produces immediate matrix disruption resulting in cell death at the impaction site and further radial progression of cell apoptosis to non-impacted area of the HAC [1, 13]. This is demonstrated also in the spontaneous OA of the knee, at the specific sites of cartilage damage [14]. Cell death results in activation of caspase [15], depleting the cartilage of the cells that are able to repair the matrix. Resident chondrocytes are activated to produce inflammatory cytokines, such as IL-1ß, IL-6, TNF-α [16]. These cytokines participate to recruitment of inflammatory cells, and activation of intracellular pathways promoting the expression of metalloproteinases [MMPs −1, −3, −9, −10, −13 and aggrecanases (ADAMTs)] activating procollagenase [10, 17] and cathepsin K [18], lastly degrading proteoglycans (PGs) and type II collagen, in the cartilage matrix. Further mechanical stress on the weakened cartilage matrix produces progressive loss of the HAC [13, 19], and the release of extracellular matrix molecules in the synovial fluid (SF). In PTOA these molecules included type II collagen fragments, fibronectin, fibromodulin, hyaluronan and lubricin. As fibronectin activates the complement cascade, collagen fragments up-regulated MMPs in chondrocytes via MAPK38 and Nf-kB signalling [20, 21], further switching the cartilage from the anabolic to the catabolic state.

Considering animal models of PTOA, racehorses sustaining repetitive impact joint injuries, during their athletic career, are likely to develop DJD shortly after the initial inflammatory event affecting the joints [2, 4, 6, 7]. This progression was bound to occur at a much faster rate than in humans [22, 23], although there are currently not enough investigations and consensus about this statement.

Assaying blood, urine or SF biochemical markers could reflect qualitative and/or quantitative changes within the joint [22, 23], providing information on disease activity in the joint compartment. Biomarkers reflecting type II collagen degradation (i.e. CTXII) are currently considered the most promising index of MMPs activity, both in human and animal models of OA [24, 25]. Non-collagenous proteins like complex oligomeric matrix protein (COMP), released into the SF during HAC degradation [26], may also indicate an increase matrix turn-over in the HAC in response to exercise [27, 28], making difficult to discriminate between normal and OA-affected patients. In most cases a substantial overlap with controls indicates that a single biomarker alone may have limited diagnostic potential [29], preferring the combination of different biomarkers for assessing disease activity on the individual level [30, 31]. Practical application of biomarkers assessment was found very promising in racehorses, where they could indicate the pattern of disease activity (inflammatory *versus* degradative status of the joint) during PTOA and verifying treatments efficacy like in other animal models [32], but they continue to be of limited use due to the insufficiency of longitudinal studies.

### Objective of the study

As a primary objective, this study will compare kinetics of structural biomarkers and pro-inflammatory cytokines in the SF and bloodstream of a group of racehorses with fetlock joint injury *versus* a group of controls, monitored during 5 years of racing career from the early stages of disease onset. Considering only the PTOA group, this study will:

1. Evaluate the reliability of a combination of pro-inflammatory cytokines (IL-1ß, IL-6, and TNF-α) and cartilage degeneration biomarkers (COMP and CTXII) to define disease activity and predict PTOA progression in comparison to radiographic assessment of the joints;
2. Assess the relationship between serum and SF biomarkers at the same time-point;
3. Investigate the relationship between the inflammatory status and the HAC degradation in the fetlock joints affected by PTOA, during active training, in order to modulate joint therapy.

### Hypothesis of the study

We hypothesised that repetitive biomarker immunoassays, during active training, could confirm progressive degeneration in PTOA-affected joints following a spontaneous joint injury, in comparison to healthy controls. Furthermore, in evaluating the inflammatory and/or degradative status of the HAC we would be able to characterize disease activity, and define the phenotype of PTOA during disease progression. Lastly, our hypothesis was that targeting pro-inflammatory cytokines, in combination to training suspension, would be effective in PTOA prevention.

## Methods

### Animals and experimental design

This is a longitudinal and cross-sectional cohort study. Horses enrolled in this study were part of a larger cohort of animals monitored from September 2008 to August 2013 and previously described [33]. Racehorses younger than 3-years old experiencing a spontaneous traumatic fetlock OA were assigned to the PTOA group at the moment of the lesion (T0). The diagnostic criteria for diagnosing traumatic fetlock OA were a significant clinical improvement of the lameness score after intra-articular (IA) diagnostic analgesia or low meta-carpal/tarsal nerve block, and the presence of one of the following findings: osteochondral fragmentation at the proximal and dorsal border of the proximal phalanx, detection of osteophytes and/or entesiophytes at the articular margins at the level of the capsular insertions, ultrasonographic signs of chronic synovitis, and signs of subchondral bone trauma at the magnetic resonance imaging. Animals with developmental osteochondral pathologies and/or major fractures affecting the target joints were excluded from the study. For each PTOA horse included in the study, a control subject from the same training centre was enrolled concomitantly. Control horses (C group) were randomly selected amongst age-matched STBRs, which were sound at the time of their physical examinations and radiographically normal. In each control it was selected a fetlock joint with the criterion of respecting the distribution between front- and hindlimbs observed in the studied horses. Animals in both groups, which sustained major musculoskeletal injuries and were retired before the completion of the study were excluded from the cohort. Likewise, animals that received IA corticosteroids in the targeted joints and animals tested positive for doping were ruled out. All joint medications administered to racehorses included in the PTOA-group during the study period complied with the local anti-doping agency guidelines^1^. Racehorses in the PTOA-group received a personalized joint treatment immediately after the acute trauma with a simple training suspension, and IA joint medication alone, or a combination of arthroscopic surgery and joint medications associated to training suspension during the period of joint rehabilitation.

**Fig. 1:**
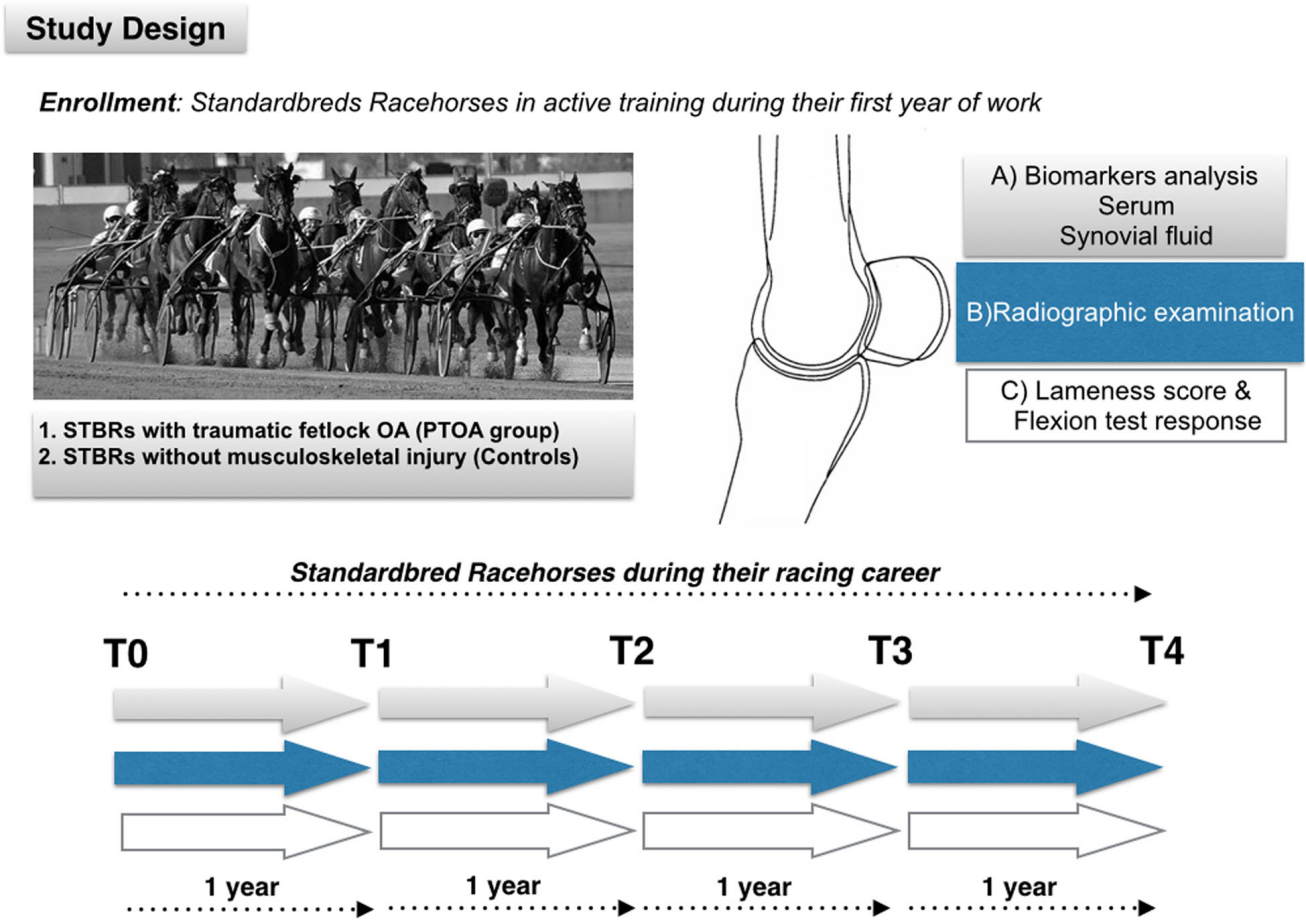
Schematic design of the study

Since their enrolment in the study, the animals were strictly monitored to assess the progression of degenerative changes in the target joints. Specifically, they underwent yearly clinical and radiographic examination, as well as blood and synovial fluid sampling for five consecutive years (Fig. 1). All the enrolled racehorses underwent regular training during throughout the study period.

### Clinical and radiographic assessment of the joint

Horses were observed trotting on a straight line (hard surface) to assess lameness severity, before and immediately after distal limb flexion tests. Video-recordings were taken of each clinical examination and blindly analysed by one operator, unaware of the horse ID, at the end of the study. Lameness was marked with 0 to 5 points as advised by the American Association Equine Practitioner [34] (Table 1). The response to the flexion tests was marked 0 to 3 in relation to the increase in lameness score of the animal (where 0 = negative response and 3 = markedly positive response).

**Table 1:** Lameness score adopted in our study (AAEP score)

Score 0	= sound in all circumstances (walk, trot and fast work at the racetrack)
Score 1	= lameness difficult to detect and not consistently obvious, regardless of circumstances^a^
Score 2	= lameness undetectable at walk and difficult to detect at trot in straight line, but consistently apparent under certain circumstances of fast work^a^
Score 3	= lameness consistently detectable at trot in straight line and under all circumstances
Score 4	= obvious lameness at walk
Score 5	= minimal weight bearing

^a^all racehorses with low-grade lameness scores were observed during fast work at the racetrack for proper assessment of 1/5 and 2/5 lameness scores

A semi-quantitative radiographic score ranging from 0 to 33 points (Table 2), modified from the one previously adopted by Cleary [27], was used to assess radiographic severity of OA. The radiograms were anonymous with regard to the patients’ data and chronology, and were evaluated blindly. Two experienced clinicians defined the radiographic scores by consensus.

**Table 2:** Radiographic score adopted to assess fetlock joint OA in horses based on standard x-ray examinations

Radiographic parameters^a^	Score
Joint space narrowing	0 to 3
Osteophytes number	0 to 3
Osteophytes size	0 to 3
Enthesiophytes number	0 to 3
Enthesiophytes size	0 to 3
Subchondral bone changes at the palmar/plantar aspect of the metacarpal/metatarsal condyles	0 to 3
Osteochondral fragmentations number	0 to 3
Osteochondral fragmentations size	0 to 3
Soft tissues swelling	0 to 3
Supracondylar bone resorption	0 to 3
Proximal sagittal crest osteolysis	0 to 3

^a^all parameters were scored 0 (normal) to 3 (pathologic) and summed up together

### Biological sample processing

Blood and SFs were collected during the first lameness investigation (T0) in the PTOA group and were then repeated during four consecutive years (T1 to T4). In the C group, SFs were collected at T0 randomly from one of the fetlock joints, and later assessments were repeated in the same joint. Synovial fluid was taken aseptically from the proximal palmar/plantar pouch of the fetlock joints with the joint slightly flexed, collected in EDTA-containing tubes and placed on ice. Blood was obtained from jugular venepuncture using EDTA-tubes and placed on ice. Before each sampling, the horses were housed in a stall for 2 consecutive days and they were not exercised. Within 1 h from collection, the blood was centrifuged at 3500 g x for 12 min at 4 °C. Serum aliquots (5x1 ml) were collected in plain tubes and were stored at −80 °C. Synovial samples were centrifuged at 3200 g x for 10 min at 4 °C to remove cells and debris and were mixed with 1 mM phenylmethylsulphonylfluoride (PMSF, an inhibitor of serine proteases), and 5×1-ml aliquots were stored at −80 °C. Before the ELISA tests, the SF was treated with hyaluronidase (Hyaluronidase from bovine testes, Sigma H3884, Sigma-Aldrich, Saint Louis, Missouri, USA) at a concentration of 20 UI/ml for 30 min at 37 °C to reduce their viscosity and were diluted 1:2 with HPE-0.1375 % Tween buffer solution (Sanquin Reagents, Amsterdam, Netherlands).

### ELISA tests

Interleukin-1ß concentrations in the collected samples were determined using a commercial direct ELISA kit (ABIN572441, antibodies-online Inc., Atlanta, Georgia, USA) with a rabbit polyclonal antibody raised against equine IL-1ß. Interleukin-6 concentrations were measured with a commercially available sandwich ELISA kit using a mouse monoclonal antibody raised against equine IL-6 (SEA079Eq, USCN Life Science Inc., Wuhan, China). Tumour necrosis factor-α concentrations were measured with a direct ELISA kit (bs-2081R, Bioss Inc., Woburn, Massachusetts, USA) using a rabbit anti TNF-α polyclonal antibody. Cartilage oligomeric matrix protein concentrations were determined with a commercially available sandwich ELISA kit (MBS006795, MyBioSource Inc., San Diego, California, USA) using a monoclonal antibody against the equine COMP. Cross-linked C-telopeptide fragments of type II collagen concentrations were assessed with a sandwich ELISA kit (code AC-08 F1, Serum Pre-Clinical Cartilaps, IDS, Boldon, UK) using a monoclonal antibody raised against CTXII fragments (6 amino acids of the sequence EKGPDP) as described previously. All of these assays had been previously validated for use with equine serum and SF [27, 35–37]. Absorbance was measured at 450 nm with a microplate reader. Each sample was analysed in duplicate. Positive, negative and blank controls were included on each plate in duplicate. The intra- and inter-assay coefficients of variability (%CV) were calculated for each series of assays.

### Statistical analysis

Values are expressed as median and 95 % C.I. Continuous variables were tested for normality with the Shapiro-Wilk normality test. Abnormally distributed variables underwent Log10 transformation. Effect of time and group on biomarker levels were studied with two-way ANOVA and Bonferroni post-tests for multiple comparisons.

Considering only the PTOA group, a mixed regression model was employed to analyse how biomarkers, radiographic score and clinical scores were associated during the time of the study, with time and subjects as fixed effects. A general linear model was used to investigate how each biomarker independently contributed to explain radiological progression in the same group of animals.

Correlations between SF and the serum concentrations of each biomarker, at different time points, were evaluated using Pearson correlation tests in the PTOA group. Correlations between different biomarkers at each time point were calculated using Spearman correlation tests to investigate the associations between pro-inflammatory cytokines and structural biomarkers of HAC degradation. *P*-values <0.05 were considered statistically significant.

## Results

Thirty-seven animals fulfilled the inclusion criteria for the PTOA group during the study period. However, 9 racehorses sustained orthopaedic injuries preventing further training and were retired, and 3 animals received corticosteroids IA, causing their exclusion from the study. Finally, only 25 racehorses were included in the PTOA group. Among the control horses, of the 37 enrolled initially, only 30 fulfilled the inclusion criteria, but only 16 animals completed the 5-years period of training studied. In the PTOA group only 21 % of the horses received an arthroscopic surgery to remove an osteochondral fragmentation associated to the acute joint trauma, as 78 % of the horses received multiple joint medications, using autologous Interleukin-1 Receptor Antagonist Protein (IL-1Ra) or hyaluronic acid. IL-1Ra treatments were prepared according to the instructions of the manufacturer (Orthokine Vet IRAP 60, DE). All horses that had a joint injury were initially managed with a reduced exercise regimen in addition to IA medication or surgery (arthroscopy) and a variable period of box-rest and rehabilitation (i.e. walking, swimming, or water-treadmill from 6 to 12 weeks after surgery and from 1 to 3 weeks after joint medication) before to gain progressive training. All the animals were sound when they were back in training. Clinical details of the horses studied are summarized in Table 3.

**Table 3:** Baseline clinical characteristics of racehorses at the time of inclusion and during the study period

	PTOA-group (*n* = 37)	Healthy subjects (*n* = 37)
Standardbred racehorses with at least 6 months of training at the time of inclusion, no. (%)	35 (94 %)	37 (100 %)
Gender, no. (%) male/no. (%) female/no. (%) gelding/no. (%)	16 (43 %)/12 (32 %)/9 (24 %)	11 (29 %)/16 (43 %)/10 (27 %)
Age at the moment of the enrolment (months)	32 (27–35)	34 (29–35)
Disease duration, referring to the lameness identification (days)	5 (2–19)	–
Administration of anti-inflammatory therapy before diagnosis, no. (%)	5 (14 %)	–
Lameness score at clinical examination (0–5 scale)	2 (2–3)	0
Flexion test response in the affected leg at clinical examination (0–3 scale)	2 (1–3)	0
Horses sound after intra-articular analgesia with mepivacaine, no. (%)	22 (61 %)	–
Horses sound after low 4-points-metacarpal (6-points-metatarsal) nerve block, no. (%)	15 (39 %)	–
Radiographic score of the affected fetlock joint at T0 (0–33 scale)	5 (2–11)	0
Osteochondral fragmentation at the dorsal border of the proximal phalanx, no. (%)	9 (24 %)	–
MRI findings of subchondral bone trauma in the metacarpal/metatarsal epiphysis or subchondral bone of the proximal phalanx, no. (%)	3 (8 %)	–
Horses with haemarthrosis in the synovial fluid at clinical presentation, no. (%)	4 (11 %)	–
US findings of fetlock joint synovitis (thickening of the dorsal plica, increased synovial fluid)	29 (76 %)	–
Arthroscopic surgery to remove osteochondral fragmentation, no. (%)	8 (21 %)	–
Animals treated intra-articularly^a^, no. (%)	28 (76 %)	–
Animal treated intravenously or intramuscularly^b^, no. (%)	8 (21 %)	–
Days of box-rest after traumatic fetlock OA, before to gain training	42 (28–198)	
Animals retired because additional muscoloskeletal injuries or other medical problems during the study period, no. (%)	9 (24 %)	17 (46 %)
Animals exluded because they were positive at doping controls/received corticosteroids intra-articularly, no. (%)	3 (8 %)	4 (11 %)
Animals that completed the time-course of the study, no. (%)	25 (68 %)	16 (43 %)

*Abbreviations*: *MRI* magnetic resonace imaging, *US* ultrasound

^a^Drugs administered intra-articularly were: Interleukin-1-Receptor-Antagonist-Protein (IL-1Ra), hyaluronate, polysulfated glycosaminoglycan

^b^Drugs administered intramuscularly or intravenously were: Tiludronate (IV or IM) and polysulfated glycosaminoglycan (IV)

Except where indicated otherwise, values are the median (range or percentage in brackets) in all the patients

### Clinical evaluation of lameness in the PTOA vs. C groups

In the C group, all of the horses were clinically sound and did not respond to flexion tests during the whole study period. In the PTOA group, the lameness score was highest initially, returned to baseline value at T1–T3, but increased again at T4. Lameness scores at T4 were significantly higher than at T1, T2, and T3 (Table 4). The flexion test responses at T0 were greater than at T1, T2, and T3. Also, the flexion test response was greater at T3 than at T2, and at T4 than at T1, T2, and T3 (Table 5).

**Table 4:** Average Lameness score over 5 years of the study, in the Control and PTOA groups

	PTOA group (*n* = 25)	Control group (*n* = 16)
T0	2.0 (2.0–3.0)^a^	0.0 (0.0–0.0)^b^
T1	0.0 (0.0–0.0)^b^	0.0 (0.0–0.0)^b^
T2	0.0 (0.0–0.0)^b^	0.0 (0.0–1.0)^b^
T3	0.0 (0.0–1.0)^b^	0.0 (0.0–0.0)^b^
T4	1.0 (1.0–2.0)^a^	0.0 (0.0–1.0)^b^

Values are reported as median with the corresponding 25 % and 75 % percentile (parenthesis). Comparison intra- and inter-groups are shown. Superscript letters that are different show a significant difference (*p*-value <0.05) between groups for this outcome variable

**Table 5:** Average Flexion tests response over 5 years of the study, in the Control and PTOA groups

	PTOA group (*n* = 25)	Control group (*n* = 16)
T0	2.0 (1.0–3.0)^a^	0.0 (0.0–0.0)^b^
T1	0.0 (0.0–1.0)^b^	0.0 (0.0–1.0)^b^
T2	0.0 (0.0–1.0)^b^	0.0 (0.0–0.0)^b^
T3	1.0 (1.0–1.0)^c^	0.0 (0.0–1.0)^b^
T4	2.0 (1.0–2.0)^a^	0.0 (0.0–0.0)^b^

Values are reported as median with the corresponding 25 % and 75 % percentile (parenthesis). Comparison intra- and inter-groups are shown. Superscript letters that are different show a significant difference (*p*-value <0.05) between groups for this outcome variable

### Radiographic evaluation in the PTOA vs. C groups

**Fig. 2:**
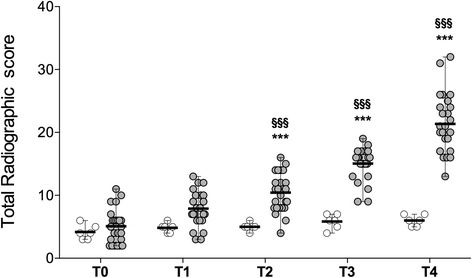
Total radiographic score of the fetlock joints over 5 years of the study in the Control and PTOA groups. Data are presented as mean, and 95 % confidence interval. *P*-values were determined by comparisons of the groups at any time point and in the groups, against the value at T0, with two-way ANOVA. Stars (*) represent significant differences between the 2 groups at the same time point (*:*p* < 0.05; **:*p* < 0.01; ***:*p* < 0.001). Symbols (§) represent significant differences with T0 in the same group (§:*p* < 0.05; §§:*p* < 0.01; §§§:*p* < 0.001)

In the C group, the radiographic scores remained stable throughout the study period. In the PTOA group, the radiographic scores were lower at T0 compared to T2, T3, and T4 (Fig. 2).

### Synovial fluid and serum biomarker concentrations in the PTOA vs. C groups

**Fig. 3:**
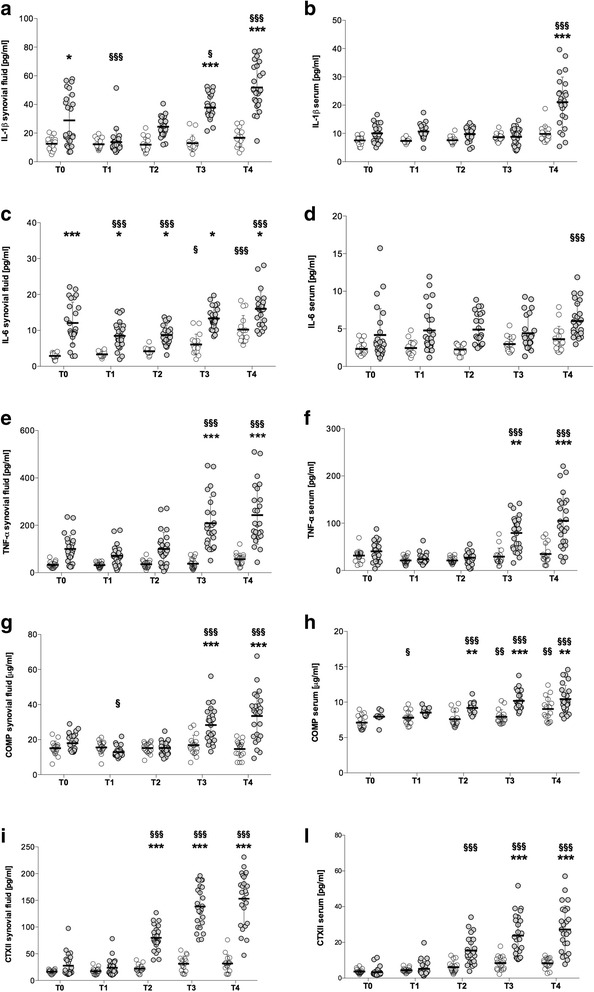
Values of inflammatory cytokines and structural biomarkers in synovial fluid and serum. Healthy controls (C group; *n* = 16) and racehorses with traumatic osteoarthritis at the fetlock joint (PTOA group; *n* = 25) are reported during the timeframes of the study (T0 to T4). Values in synovial fluid are shown in panels **a**, **c**, **e**, **g**, and **i**. Values in serum are shown in panels **b**, **d**, **f**, **h**, and **l**. Data are presented as median, and 25^th^ to 75^th^ percentiles (whiskers). *P*-values were determined by comparisons of the groups at any time point and in the groups at different time points with two-way ANOVA. Stars (*) represent significant differences between the 2 groups at the same time point (*:*p* < 0.05; **:*p* < 0.01; ***:*p* < 0.001). Symbols (§) represent significant differences with T0 in the same group (§:*p* < 0.05; §§:*p* < 0.01; §§§:*p* < 0.001)

Biomarkers concentrations were always within the minimal detection values of the immunoassays. An intra-assay %CV < 10 % and an inter-assay %CV < 15 % were considered acceptable. A significant effect of both group (PTOA vs. C) and time was identified for the synovial concentrations of IL-1β (*p* < 0.0001), IL-6 (*p* < 0.0001), TNF-α (*p* = 0.0025 and *p* < 0.0001), COMP (*p* = 0.001 and *p* = 0.0001), and CTXII (*p* < 0.0001) (Fig. 3). The concentrations of IL-1β, TNF-α, COMP, and CTXII in SF did not vary with time in the C group, demonstrating that age and exercise *per se* do not affect the SF concentrations of these biomarkers in healthy horses. Only IL-6 concentrations significantly increased with time in the SF of the C group, with the values detected at T3 and T4 significantly higher than those detected at T0 (*p* < 0.05 and *p* < 0.001, respectively).

In the PTOA group the effect of acute joint injury induced a significantly increase in SF levels of IL-1β and IL-6 at T0, which persisted at T1, only for IL-6. Levels of IL-1β in PTOA-affected joint returned to pre-injury levels 1 year after the acute joint trauma. At T2, levels of IL-6 and CTXII in SF were significantly elevated in the PTOA-affected joints compared to exercise-alone joints of the C group. At T3, concentration of IL-1β, IL-6, TNF-α, COMP and CTXII in SF were all significantly elevated in the PTOA-affected joints compared to C group and these difference persisted also in the last measurement.

As for serum samples, a significant effect of both group (PTOA vs. C) and time was noticed on the concentrations of IL-1β (*p* < 0.0001), IL-6 (*p* = 0.04 and *p* = 0.03), TNF-α (*p* = 0.0002 and *p* < 0.0001), COMP (*p* = 0.0002 and *p* < 0.0001), and CTXII (*p* = 0.0025 and *p* < 0.0001) (Fig. 3). The serum concentrations of IL-1β, IL-6, TNF-α, COMP, and CTXII did not vary over time in the C group, proving that age and exercise *per se* do not affect the concentrations of these biomarkers in healthy horses. There was no increase in pro-inflammatory cytokines in serum of PTOA-affected horses compared to exercise alone horses at T0, T1 and T2. Only at T4, levels of IL-1β in serum were elevated in PTOA-affected animal compared to C horses. There were no significant increases in serum levels of IL-6 in horses with PTOA, during all the study. TNF-α level in serum started to rise significantly in PTOA-affected animals at T3 and differences persisted at T4, similarly to the values of CTXII. In PTOA group the effect of disease induced a significantly rise in serum levels of COMP at T2, which persisted throughout the duration of the study.

### Correlation between synovial fluid and serum biomarkers

There were significant correlations between SF and the serum levels of IL-1β in horses with PTOA, only at T4. The mean level of IL-1β in the SF of PTOA affected joints compared to serum, for the same horses, was 2.5-fold higher. As for IL-6, its serum concentration reflected SF levels at T0 and T4 only in the PTOA group, and the mean level of IL-6 were 2.8-fold and 2.6-fold higher in the SF compared to the serum, respectively. CTXII levels in serum reflected SF levels in PTOA-affected joint at T3 and T4, and the mean levels of CTXII were 5.8-fold and 5.6-fold higher in the SF compared to serum, respectively. An exhaustive summary of the results of correlation tests between serum and SF biomarkers at different time-points is provided in Table 6.

**Table 6:** Synovial Fluid and Serum correlations at different time-points

Synovial fluid/Serum concentration		T0	T1	T2	T3	T4
PTOA group	IL-1b	–	–	–	–	*r* = 0.43*
	IL-6	*r* = 0.43*	–	–	–	*r* = 0.51**
	TNF-a	–	–	–	*r* = 0.53**	–
	COMP	–	–	–	–	–
	CTXII	–	–	–	*r* = 0.57**	*r* = 0.63***
C group	IL-1b	–	–	–	–	–
	IL-6	–	–	*r* = −0.85*	–	–
	TNF-a	–	–	–	*r* = 0.87*	–
	COMP	–	–	–	–	–
	CTXII	–	–	–	–	–

Correlations of pro-inflammatory cytokines and structuralbiomarkers values, in the synovial fluid and serum, at different time-points, in both the PTOA and C groups, over the 5 years of the study. P-values and coefficient of regression (r) are shown. Significant correlations are represented as bolded R-values with the superscript star. Key: *:*p* < 0.05;**:*p* < 0.01;***:*p* < 0.001

### Temporal changes in pro-inflammatory cytokines and structural biomarkers during training and correlations with radiographic and clinical scores

**Fig. 4:**
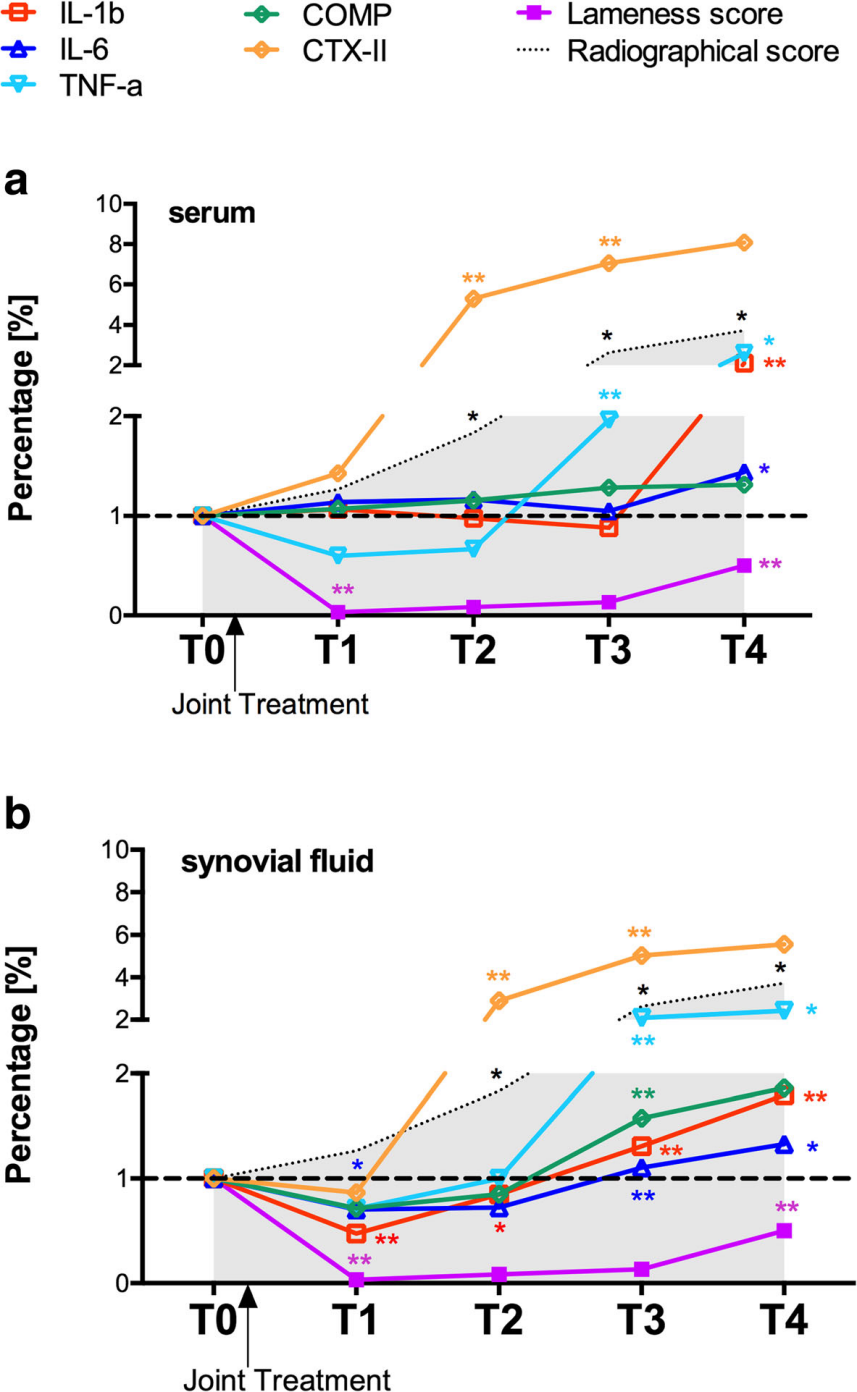
Temporal course of OA biomarkers levels and corresponding clinical scores in the PTOA group. Percentual variations of OA biomarker levels and corresponding radiographic and lameness scores, in the PTOA group of racehorses (*n* = 25), during 5 years of racing activity. Panel **a** refers to values in the sera. Panel **b** refers to the synovial fluid values. Data for each time point represent the mean levels of IL-1ß, IL-6, TNF-α, CTXII, COMP, Radiographic score and Lameness score. Standard deviation (SD) error bars are not plotted in these graphs for clarity. Reference value at T0 is delineated (dotted line). *p < 0.05 *versus* level at previous time-point. **p < 0.001 *versus* level at previous time-point

All data from T0 to T4 in PTOA group were transformed and expressed as percentage change compared to the value of the same variable at the previous time-point (Fig. 4). In the early stage of PTOA, SF level of pro-inflammatory cytokines decreased in the first year of training, following joint medications or arthroscopic surgery (IL-1β and IL-6 at T1 were significantly lower than each baseline level). As expected, indices of OA disease activity increased following the acute joint injury and became significantly elevated at T2 (CTXII) and at T3 (COMP), gradually increasing until the end of the study. Interestingly, levels of pro-inflammatory cytokines increased significantly starting from T2 (IL-1β) to T3 (IL-6, TNF-a) and demonstrated a quite similar pattern that CTXII and radiological score, meaning that structural joint damage, particular in term of cartilage destruction is associated with the degree of joint inflammation, during the progression of PTOA. Of note, the lameness score increased only after the structural damage and the joint inflammation progressed for many years, at the end-point of the study. The pattern of the pro-inflammatory cytokines and the structural biomarkers in serum appeared to synchronize to SF changes, starting from T2. Only level of COMP in serum remained unchanged during 5 years.

Correlations between the variation of a single biomarker between 2 years (i.e. T0-T1, expressed as Δ_T0-T1_) and the variation of radiographic scores between the subsequent 2 years (i.e. T1-T2) are shown in Table 7. In the SF, the percentage increase of TNF-α level at T3 vs. T2 was significantly correlated with the percentage increase of the radiographic score at T4 vs. T3. Moreover, a trend to significance was identified between the percentual variation of synovial TNF-α at T2 vs. T1 and the percentual variation of radiographic score at T3 vs. T2. In serum, the percentage variation of IL-6 at T3 vs. T2 was significantly correlated with the percentage change of the radiographic score at T4 vs. T3. No others significant correlations were noted between the percentage variation of biomarkers levels in SF and serum and the percentage changes of the clinical scores in the PTOA group.

**Table 7:** Correlation between biomarkers percentual change (Δ) and percentual change of radiological score (Δ) in the subsequent year, in PTOA affected horses only

		Δ mean radiographical score of T _(x+1)_−T _(x)_		
		T _(x)_ = T1	T _(x)_ = T2	T _(x)_ = T3
ΔIL-1b of T _(x)_−T _(x−1)_	Serum	–	–	–
	Synovial Fluid	–	–	–
ΔIL-6 of T _(x)_−T _(x−1)_	Serum	–	–	***r*** ** = 0.42 **(***p*** ** = 0.041**)
	Synovial Fluid	–	–	–
ΔTNF-a of T _(x)_−T _(x−1)_	Serum	–	–	–
	Synovial Fluid	–	***r*** ** = 0.38** (***p*** ** = 0.058**)	***r*** ** = 0.435** (***p*** ** = 0.030**)
ΔCOMP of T _(x)_−T _(x−1)_	Serum	***r*** ** = 0.39** (***p*** ** = 0.053**)	–	–
	Synovial Fluid	–	–	–
ΔCTXII of T _(x)_−T _(x−1)_	Serum	–	–	–
	Synovial Fluid	–	–	–

Significant correlations are represented in bold

### Association between pro-inflammatory cytokines and structural biomarkers in the serum and synovial fluid of the PTOA group

Significant correlations were observed among various biomarker in the serum and SF at different time-points (Table 8). Specifically, significant correlations between pro-inflammatory cytokines were detected: IL-1β to IL-6 at T0, both in serum and SF, IL-1β to TNF-α at T3 and T4, only in the SF, and IL-6 to TNF-α at T3, only in serum. Interestingly, correlations between structural biomarkers and pro-inflammatory cytokines were observed. Strong significant correlations were noted between CTXII and TNF-α in the SF, both at T3 and at T4, and a moderate correlation at T0. Moderate significant correlations were observed for the IL-1β and COMP in the SF, at T0, T1, T3 and T4. Similarly, significant correlations were observed between IL-6 and CTXII in the SF, only at T2 and between IL-6 and COMP, at T0, both in serum and SF, and at T2 and T4, only in serum. No significant correlations were observed between structural biomarkers.

**Table 8:** Correlation between different cytokines and structural biomarkers in the PTOA affected race horses

		IL-1ß					IL-6					TNF-α					COMP					CTX-II				
		*T0*	*T1*	*T2*	*T3*	*T4*	*T0*	*T1*	*T2*	*T3*	*T4*	*T0*	*T1*	*T2*	*T3*	*T4*	*T0*	*T1*	*T2*	*T3*	*T4*	*T0*	*T1*	*T2*	*T3*	*T4*
IL-1ß	*S*	1.0	1.0	1.0	1.0	1.0	**0.40**	–	–	–	–	–	–	–	–	–	–	–	–	–	–	–	–	–	**0.41**	–
	*SF*	1.0	1.0	1.0	1.0	1.0	**0.69**	–	–	–	–	–	–	–	**0.68**	**0.58**	**0.66**	**0.50**	–	**0.48**	**0.45**	–	–	–	–	–
IL-6	*S*						1.0	1.0	1.0	1.0	1.0	–	–	–	**0.43**	–	**0.53**	–	**0.41**	–	**0.52**	–	**0.42**	–	–	–
	*SF*						1.0	1.0	1.0	1.0	1.0	–	–	–	–	–	**0.61**	–	–	–	–	–	–	**0.58**	–	–
TNF-α	*S*											1.0	1.0	1.0	1.0	1.0	–	–	–	–	–	–	–	–	–	–
	*SF*											1.0	1.0	1.0	1.0	1.0	–	–	–	–	–	**0.51**	–	–	**0.69**	**0.71**
COMP	*S*																1.0	1.0	1.0	1.0	1.0	–	–	–	–	–
	*SF*																1.0	1.0	1.0	1.0	1.0	–	–	–	–	–
CTXII	*S*																					1.0	1.0	1.0	1.0	1.0
	*SF*																					1.0	1.0	1.0	1.0	1.0

Only the coefficients of (r) of significant groups are shown in bold, for clarity

## Discussion

This study demonstrates that an acute episode of traumatic OA, in the first year of training, is able to induce an accelerated progression of PTOA in the affected fetlock joints, from a normal state to an end-stage disease, within 4 years. This is the first study in which the long-term consequences of an impact joint injury have been evaluated in STBRs with spontaneous PTOA, regularly trained after a rehabilitation period, and using biomarkers to assess disease activity. Previous studies focused on OA biomarkers in experimental horse models spread their observations to a limited number of weeks following the acute joint injury [2, 7, 27, 38–40].

Osteoarthritis progression was not characterized by linearity, demonstrating that early inflammation and structural changes in the HAC are not ever associated, and they could be effectively modulated by the intensity of training and therapeutic management. Traumatic OA is characterized by a peak of pro-inflammatory cytokines in the SF, as previously reported [16, 41]. Despite this finding, 1 year after the impact-injury, there were no radiographic and biochemical signs of joint disease in the fetlock joints that sustained traumatic injuries, and signs of lameness subsided. At this stage, PTOA-affected animals were all undergoing new bouts of repetitive exercise. Our features confirmed that joint injury at a young age decreases the tolerance of the cartilage matrix to the increased magnitude of mechanical stress, as new bouts of workloads are associated with structural damage of the HAC. Structural changes in the HAC of the PTOA-affected joints, based on CTXII level in the SF, started rise 2 years after the acute joint trauma. This corresponds to the age of 4 years old, when those racehorses joined the higher speed at races. Previous studies in animal models provided evidence that damage to the collagen network of the HAC is an early event occurring concurrently to the acute joint injury [22]. Also, an experimental model of OA in Thoroughbred racehorses showed an increase in aggrecan (CS846-epitope), COMP, and collagen type II fragments (C1, 2C), both in serum and SF, which were able to discriminate horses with OA vs. exercise-alone animals, at 12 weeks following joint trauma [2]. However, those studies were based on higher destructive models of OA than in our study: only a small percentage of the horses in our study cohort sustained an osteochondral fragmentation, all the animals of this group were rested after the onset of lameness, and exercise was suspended for a longer period of time than in experimental studies, before to return at regular training.

Recent researches pointed out on the role of mechano-trasduction pathway throughout the extracellular matrix, which has a causal role in triggering OA. Biochemical changes could affect the mechanical properties of HAC: lysyl-oxidase (LOX) pathway increases cross-linking in the collagen substrate and cartilage stiffness, LOX overexpression affects chondrocytes activation profiles of transcriptional factors, and among these the NF-kβ pathway, which is associated to the increased expression of matrix-degradative enzymes [42]. Modification in the pericellular matrix in HAC has a critical role in controlling the module of elasticity of the cartilage [43]. Chondrocytes from OA cartilage showed an altered response to mechanical stimulation, with an aberrant cellular signalling leading to increased cartilage breakdown [44].

Structural biomarker levels, during progression of joint disease, became associated with the increased level of pro-inflammatory cytokines, when we identified more pronounced features of inflammation. Cartilage degradation products, that are released into the SF, might induce cytokines independent MMP-induction [45] and an increase of pro-inflammatory cytokines [20]. Also COMP, released during cartilage degradation, was able to activate complement in the SF [46]. Furthermore, experiments with chondrocyte cultures have clearly demonstrated that IL-1ß and TNF-α increase cleavage of collagen type II during OA [47].

These finding are in accordance with the observation that targeting pro-inflammatory cytokines following joint injury prevents PTOA in a mouse model of the disease [32, 48, 49], like in ankylosis spondylitis [50], and rheumatoid arthritis [51, 52] . We employed IL-1Ra, via intra-articular pathway, to antagonize the main pro-inflammatory cytokine, early after acute joint trauma. Early and targeted treatment was effective to reduce pro-inflammatory cytokines levels in the SF of affected joints and to control cartilage degradation in the joint tissue along an entire year. This finding raised the question if the inflammatory status had been maintained in the targeted joints, when pro-inflammatory cytokine levels in the SF normalized. Inflammatory cytokines may be expressed at higher levels in diseased cartilage [53], and at the level of the subchondral bone [54], and synovial membrane [16] than found in evaluating the SF, as it was detected in humane hip OA [53].

We are unable to drawn any conclusion about the effectiveness of our therapeutic strategies during PTOA at long-term, since the ability of such treatments to modify disease progression is still limited.

The rapid decline of joint health, which was highlighted in our study in the PTOA-affected racehorses, caused an increase of joint pain and a consequent lameness. The contribution of the pro-algesic effect of cytokines had been recently pointed out. TNF-α and IL-1β can contribute to hyperexcitability of small-diameter unmyelinated C-fibers, that carry pain signals from peripheral tissues to the dorsal root ganglia [55], while IL-6 alone increases neuronal response to substance P [55]. Contrary to what is reported in humane knee during PTOA [56], TNF-α level does not exhibit a clear correlation with the current clinical scale of joint pain adopted to assess lameness in horses. Assessment of TNF-α in the SF was able to anticipate radiographic deterioration in the targeted joints, more than the percentual change of structural biomarkers. For this reason, suppression of TNF-α could be an intriguing prospect in equine PTOA and requires specific investigation.

Even if CTXII offers a useful marker reflecting on-going cartilage damage, it was not correlated with the radiographic score of the OA affected joints in racehorses [27]. A weak association between the radiographic parameter indicative of cartilage degradation (JSN = joint space narrowing) and CTXII level in the SF was detected only at T3 (*p* = 0.05 and *r* = 0.15; data not shown). This is not surprising given that the majority of parameters used to define the radiographic score in racehorses’ studies are related with chronic synovitis and inflammation rather than to cartilage degradation. In a terminal study in rabbits, CTXII level peaked in serum 6 weeks after experimental knee injury and subsided during later degenerative stages, when the cartilage destruction was complete and the joint space is reduce [37]. Probably, this fall in the CTXII levels was not detectable in our study, because of the less destructive PTOA version in our animal model.

A previous study in racehorses with carpal joint injury identified that IL-6 was increased in the joints in association with the presence of an osteochondral fragmentation, confirming that joint fragments themselves represent a source of inflammation [57]. Osteochondral fragments were arthroscopically removed in our study, and this could explain in part our results. Noteworthy, a significant correlation between IL-6 levels in serum and the osteophyte numbers and size, in the PTOA-affected joints, was detectable at T3 and T4 (*p* < 0.05 for both; data not shown). This observation was confirmed in another study [58], where it was detected an increased mRNA expression for IL-6 from osteophytes in the OA-affected joints [58].

In contrast to CTXII, COMP in serum was able to detect OA progression 2 years following acute joint trauma. Moreover, COMP in the SF became significantly different than at the previous time point concurrently with the increasing CTXII levels in the same medium. Clinical studies in horses obtained contrasting results regarding the temporal course of COMP levels in serum and SF in early and established OA cases [28, 59–61]. It was demonstrated a variation of COMP levels in serum 1 to 5 h after an intense exercise in healthy TBRs [28], which returned to pre-existing values by 24 h. COMP is preferentially expressed in the middle- and deep-layer of the hyaline cartilage during enhanced matrix turnover [60], but COMP fragments could not easily join the SF, when the HAC is not structurally damaged [60]. Trend of COMP in the serum could be related to the capacity of osteoblasts to generate COMP [62] during subchondral bone turnover. In our study, the SF level of COMP shows a strong correlation with the lameness score at different time-points, even if this result is difficult to explain because it is impossible to determine the tissue from which COMP originated.

Of note, there is a poor correlation between structural biomarkers in the serum and SF of the PTOA-affected joints. Only CTXII levels in the serum are correlated with SF levels at T3 and T4. This observation is in accordance with a mouse model of mono-iodoacetate induced arthritis [25], where the correlation between the biomarker values in the serum vs. SF was stronger and it was influenced by the cytokines-mediated increased permeability between compartments.

There are several limitations in the present study. First, our cohort consisted of small number of subjects with PTOA and C animals. We lacked PTOA-affected racehorses and control healthy subjects for unrelated musculoskeletal injuries, which have been retired during the timeframe of the study. Our study design was conditioned by the fact that female STBRs are frequently retired and addressed to breeding at the age of 6 years old. Further studies that include a larger cohort of animals with traumatic OA and a similar size group of normal animals would be ideal for elucidating the role of many variables during PTOA. Furthermore, more cross-sectional studies would be necessary to clarify the role of joint treatments and training to regulate OA progression. To clarify causality, evaluating more biomarkers during the progression of joint disease would be required, particularly in those animals with rapid radiographic decay. A direct assessment of HAC via whole—joint histology would provide additional information but this type of approach was not feasible in a clinical study. Alternatively, more direct assessment of the articular cartilage via MRI would have shown better correlation with the biomarker levels, but this type of imaging was not practical for repeat assessments in our patients.

## Conclusion

To our knowledge, this is the first study in which kinetics of biomarkers have been evaluated at long-term during the athletic activity of STBRs, over many years, to asses the consequences of an acute joint injury at young age. This study provided important information on biomarker interplay during the clinically relevant stages of natural joint disease in an animal model of PTOA.

The assessment of the structural and pro-inflammatory biomarkers in the SF of the affected joints reflects the progressive evolution of PTOA towards end-stage OA and provides the background to assess the effect of targeting pro-inflammatory cytokines at the early stages of naturally occurring disease. This study offers an effective prospect to incorporate biomarker assessment in clinical trials, for the objective monitoring of OA progression in racehorses. The association between the initial impact-induced cartilage damage, the treatment strategies and the progressive joint degeneration deserves further investigation.

## Abbreviations

C: Control
COMP: Complex oligomeric matrix protein
CTXII: Cross-linked C-telopeptide fragments of type II collagen
DJD: Degenerative joint disease
IA: Intra articular
IL-1ß: Interleukin-1ß
IL-6: Interleukin-6
MMP: Matrix metalloproteinase
MRI: Magnetic resonance imaging
OA: Osteoarthritis
PTOA: Post-traumatic osteoarthritis
SF: Synovial fluid
STBRs: Standardbred racehorses
TBRs: Thoroughbred racehorses
TNF-α: Tumour necrosis factor-α

## Footnotes

1 Ministero delle Politiche Agricole, Alimentari e Forestali, antidoping rules
